# Spherical Aberration and Scattering Compensation in Microscopy Images through a Blind Deconvolution Method

**DOI:** 10.3390/jimaging10020043

**Published:** 2024-02-07

**Authors:** Francisco J. Ávila, Juan M. Bueno

**Affiliations:** 1 Departamento de Física Aplicada, Facultad de Ciencias, Universidad de Zaragoza, 50009 Zaragoza, Spain; 2 Laboratorio de Óptica, Instituto Universitario de Investigación en Óptica y Nanofísica, Universidad de Murcia, 30100 Murcia, Spain; bueno@um.es

**Keywords:** blind deconvolution, microscopy imaging, scattering, spherical aberration, image restoration

## Abstract

The optical quality of an image depends on both the optical properties of the imaging system and the physical properties of the medium the light passes while travelling from the object to the image plane. The computation of the point spread function (PSF) associated to the optical system is often used to assess the image quality. In a non-ideal optical system, the PSF is affected by aberrations that distort the final image. Moreover, in the presence of turbid media, the scattering phenomena spread the light at wide angular distributions that contribute to reduce contrast and sharpness. If the mathematical degradation operator affecting the recorded image is known, the image can be restored through deconvolution methods. In some scenarios, no (or partial) information on the PSF is available. In those cases, blind deconvolution approaches arise as useful solutions for image restoration. In this work, a new blind deconvolution method is proposed to restore images using spherical aberration (*SA*) and scatter-based kernel filters. The procedure was evaluated in different microscopy images. The results show the capability of the algorithm to detect both degradation coefficients (i.e., *SA* and scattering) and to restore images without information on the real PSF.

## 1. Introduction

Confocal and multiphoton (*MP*) scanning microscopy are widespread techniques used for imaging biological tissues both in ex vivo [1,2] and living [3,4] conditions. The quality of the images acquired with these instruments is often limited by the presence of optical aberrations and scattering. Whereas the former distorts the image through the loss of high-spatial frequency contents [2,3], the latter reduces the overall contrast [5]. The combination of both leads to image degradation (i.e., reduced resolution and low contrast).

A method to assess image quality is based on the calculation of the point spread function (*PSF*) of the optical system under study. In scanning microscopy, this PSF is affected by off-axis optical aberrations such as coma or field curvature, as well as astigmatism, and spherical (*SA*) and chromatic aberrations. These can be minimised with appropriate apochromatic objectives [6], correction collars [7], deformable mirrors [2,3,8,9], or spatial light modulators [2,10].

Moreover, when thick biological samples are involved, the image quality reduces significantly at deeper locations due to an increase in both scattering and aberrations (mainly *SA*) [5,8,10,11]. Therefore, the combination of *SA* and scattering degrades the *PSF* of the microscope, limiting the penetration depth within the tissue [8,9,10,11,12]. To overcome aberrations (or to increase the penetration depth), adaptive optics (*AO*) procedures have been implemented into confocal [3] and *MP* [2,8,9,10,11,13] microscopes to compensate for these effects and provide enhanced images.

Although charaterizing light scattering effects in biological tissues is challenging, different *AO*-based tools have been reported for scattering compensation in biomedical imaging [11,14,15]. However, after the *SA* correction, residual aberrations and uncompensated scattering might be enough to degrade the optical resolution and prevent proper image analysis [16].

Deconvolution is an alternative mathematical method used in digital image processing to restore optical degradation effects [17]. However, to perform a deterministic deconvolution process, it is essential to have previous information about the operator responsible for image degradation, that is, the *PSF*.

Unfortunately, the access to this information is not always easily available. In this sense, blind deconvolution algorithms [18] allow restoring blurred images, using as a starting point an approximate geometrical distribution of the *PSF*. Blind deconvolution has been succesfully applied to restore the optical quality of images in clinical and research fields [19,20].

In this work, we present a new blind deconvolution algorithm based on an iterative-optimization process of both *SA* and wide-angle scattering *PSF* compensation. This approach can be used to restore and improve microscopy images without the need of previous information on the optical properties of the imaging systems.

## 2. Materials and Methods

### 2.1. Spherical Aberration Point Spread Function

The wave aberration (*WA*) of an optical system can be represented by the Zernike polynomial expansion. This is defined in a circular pupil, and its units can be expressed in wavelengths (*λ*) or microns. According to the widely accepted double-index convention of the Optical Society of America (now Optica) [21], this *WA*(*ρ*, *θ*) can be expressed in polar coordinates as:

(1) $$WA\left(\rho ,\theta \right)=\sum_{n=0}^{\infty }\sum_{m=-n}^{n}c_{n}^{m}\times Z_{n}^{m}(\rho ,\theta )$$

where $Z_{n}^{m}$ and $c_{n}^{m}$ are the Zernike polynomials and their corresponding coefficients, respectively. This notation uses a sub-index *n* to indicate the radial order and a super-index *m* for the frequency. In this Equation (1), *θ* ranges between 0 and 2π, and *ρ* is the normalized pupil size.

In addition, the pupil function of an optical system *P*(*ρ*, *θ*) can be expressed as

(2) $$P\left(\rho ,\theta \right)=A(\rho ,\theta )\times e^{ik\times WA(\rho ,\theta )}$$

with *A* being the amplitude and *k* = 2*π*/*λ* the wave number defined as the spatial frequency of the wave (over a specific unit distance). The squared modulus of the Fourier transform of this pupil function is the associated *PSF*:

(3) $$PSF={\left|ℑ\left[P\left(\rho ,\theta \right)\right]\right|}^{2}$$

In particular, the *SA* term of the *WA* in the Zernike expansion is defined as [21]:

(4) $${WA}_{SA}=c_{4}^{0}\times \sqrt{5\times \left(6\rho^{4}+6\rho^{2}+1\right)}$$

*SA* term has been reported to present a dominant contribution in certain research fields such as Microscopy, Astronomy or Physiological Optics, among others [6,7,8,9,10]. Altough any aberration mode can be included in the developed algorithm, previous literature has reported the benefit of compensating for this dominant *SA* aberration terms in microscopy, biomedical imaging, and physiological optics [8,9,10].

**Figure 1:**
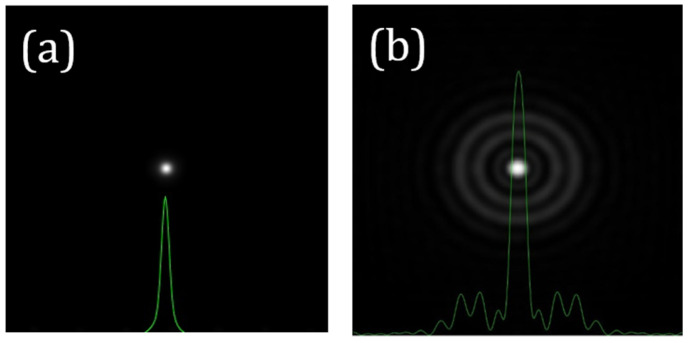
Example of computer generated *PSFs* for 0 (i.e., diffraction-limitted) (**a**) and +0.20 μm (**b**) of *SA*. The corresponding cross-sectional intensity profiles are overlayed in green. Image size: 256 × 256 μm^2^.

If the *WA_SA_* of an optical system is known, its *PSF_SA_* can be directly computed from Equation (3). As an illustrative example, Figure 1 presents the *PSF_SA_* corresponding to a *SA* value of +0.20 µm (*λ* = 800 nm and 6 mm of pupil size). This can be directly compared to a diffraction-limited PSF (i.e., no aberrations).

### 2.2. Wide-Angle Scattering PSF

Scattering effects in an optical system are usually characterized by a veiling glare distributed over the acquired image [22]. In this work, the glare spread function (*GSF*) according to the International Commission on Illumination (*CIE*) standards was used [23]. This is defined as

(5) $$\begin{array}{c} GSF\left(\alpha \right)=\left[1-0.08\times {\left(\frac{Age}{70}\right)}^{4}\right]\times \left[\frac{9.2\times 10^{6}}{{\left[1+{\left(\frac{\alpha }{0.046}\right)}^{2}\right]}^{1.5}}+\frac{1.5\times 10^{5}}{{\left[1+{\left(\frac{\alpha }{0.045}\right)}^{2}\right]}^{1.5}}\right]\\ \;\;\;\;\;\;\;\;+\left[1+1.6\times {\left(\frac{Age}{70}\right)}^{4}\right]\times \left\{\left[\frac{400}{1+{\left(\frac{\alpha }{0.1}\right)}^{2}}+3\times 10^{-8}\times \alpha^{2}\right]+PF\left[\frac{1300}{{\left[1+{\left(\frac{\alpha }{0.1}\right)}^{2}\right]}^{1.5}}+\frac{0.8}{{\left[1+{\left(\frac{\alpha }{0.1}\right)}^{2}\right]}^{0.5}}\right]\right\}\\ \;\;\;\;\;\;\;\;+2.5\times 10^{-3}\times PF \end{array}$$

**Figure 2:**
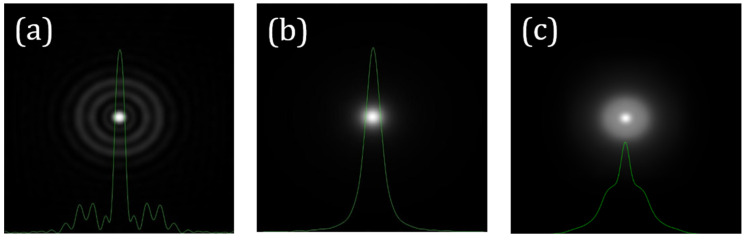
Examples of computer-generated PSFs for *SA* = +0.20 μm (**a**), *α* = 4° (**b**), and the combination of both (**c**). Similar to Figure 1, the cross-sectional profiles are also shown. PSF in (**a**) is the same as in Figure 1b. Image size: 256 × 256 μm^2^.

According to this model, the wide-angle scattered *PSF* (*GSF* or *PSF_scatt_*) depends on the veiling glare angle (*α*) and other parameters related to the imaging system [23] that are considered to remain constant within this work (*Age* and pigment factor, *PF*). Mathematically, the *PSF_scatt_* associated with an optical system in the presence of scattering is the kernel of the response during image acquisition. Figure 2b depicts an example of a *PSF_scatt_* for *α* = 4°. For a direct comparison, the *PSF_SA_* (+0.20 μm, Figure 2a) and the *PSF_SA+scatt_* combining *SA* and scattering (*α* = 4°) are also shown (Figure 2c).

### 2.3. Deconvolution Procedure

Any non-diffraction limited imaging system is affected by aberrations. In addition, if during image formation the light travels through non-transparent media, the effects of scattering are also present.

Let us suppose an imaging system with a degradation operator with a double contribution: (1) the *SA* with an associated *PSF_SA_* and (2) the scattering effects characterized by the corresponding veiling glare function (*PSF_scatt_*). If *PSF_SA+scatt_* is the *PSF* containing both contributions, the image *i′* of an object *i* through this optical system can be modelled by a convolution operation expressed as

(6) $$i^{\prime }\left(x,y\right)=PSF_{SA+scatt}⊗i\left(x,y\right)$$

The problem of obtaining the restored image *i* from this Equation (6) might be easily solved by a simple division in the Fourier domain. However, if the Fourier transform of the *PSF* contains zeros, the division would be impossible. To overpass this limitation, blind deconvolution can be used [18,19].

The blind deconvolution algorithm proposed herein is an inverse mathematical method for image restoration that assumes some information about the PSF (or the equivalent combined effect of both *PSF_SA_* and *PSF_scatt_*, i.e., *PSF_SA+scatt_*). The approach is based on the maximum likelihood estimates [24], being the first input the original image together with an initial theoretical *PSF*. Then, an iterative procedure begins, where for each Nth iteration, the estimated image ${\hat{i}}^{\left(N\right)}$ and the *PSF^(N)^* (either *PSF_SA_*, *PSF_scatt_* or the combination of both) ensures non-zero components in its Fourier transform due to the imposition of frequency band-limited constraints. These are calculated as [24]

(7) $${\hat{i}}^{\left(N+1\right)}={\hat{i}}^{\left(N\right)}\times \left(\frac{i^{\prime }}{{\hat{i}}^{\left(N\right)}⊗PSF}⊗{PSF}^{*}\right)$$

(8) $${PSF}^{\left(N+1\right)}={PSF}^{\left(N\right)}\times \left(\frac{i^{\prime }}{PSF⊗{\hat{i}}^{\left(N\right)}}⊗{PSF}^{*}\right)$$

where PSF* and *N* are the flipped *PSF* and the iteration number, respectively.

### 2.4. Algorithm Description

The algorithm here developed is divided into 2 steps. The first one (#1) is based on a through-focus sampling PSF optimization. The second one (#2) is the iterative-optimized deconvolution processing itself. Both parts are described in the following sections. The algorithm was written in Matlab (Matlab^TM^ 2021b, The MathWorks, Inc., Torrance, CA, USA).

During the image optimization process, an image quality metric is required. In particular, throughout this work, the metric “entropy” (*E*) was used. According to the theory of communication, the concept of entropy of an image can be understood as the degree of randomness of the information within an image. Therefore, *E* is an appropriate metric since it provides information on the presence of details and features within an image [25]. In previously reported iterative deconvolution approaches, image entropy has been proposed as an efficient tool to optimize the density distribution of the estimated *PSF* and restored images [26,27]. This work employs this image quality metric as a quantitative measure of the improved spatial resolution of the restored images. For its calculation, the Shannon Entropy Matlab^TM^ function was used.

#### 2.4.1. Step #1: Sampling *PSF* Optimization

**Figure 3:**
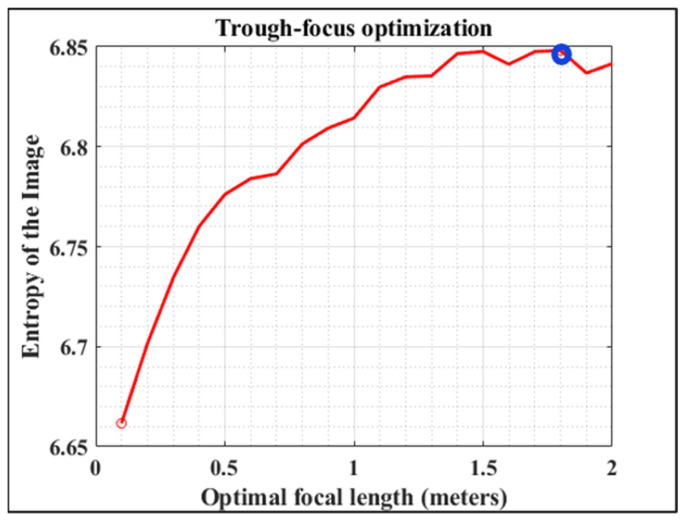
Representative example of entropy evolution as a function of *F_L_* (step #1 of the algorithm). For the sense of completeness, both minimum (red dot) and maximum (blue dot) values are marked within the plot.

This step #1 is based on an optimization process by direct search of the optimum focal length (*F_L_*) (i.e., defocus minimization) (see Figure 3). The algorithm requires the values of some initial parameters such as the number of pixels of the image (*N*), the size of each pixel (*Pixel_size_*), the wavelength (*λ*), the aperture radius (*AR*), and the ratio *F_L_*/*AR*. If the information about the optical system is completely unknown, the input parameters are exclusively reduced to provide the image size.

Then, the *SA* Zernike coefficient (C_4_^0^) is set to a negligible value (0.001 in our case). The algorithm begins with a hill-climbing searching for the optimal *F_L_* within an interval ranging between 0 and 5 m, in steps of 0.1 m. For each step, a simple blind deconvolution is performed and the E value of each deconvolved image calculated. In general, the higher the *E* value, the higher the image sharpness (i.e., more details are visualized). Once the entire closed-loop is completed, the *F_L_* value of the image with maximum E is chosen as the optimum one (Figure 3) [28].

#### 2.4.2. Step #2: Deconvolution through *PSF_SA_* and *PSF_scatt_*

**Figure 4:**
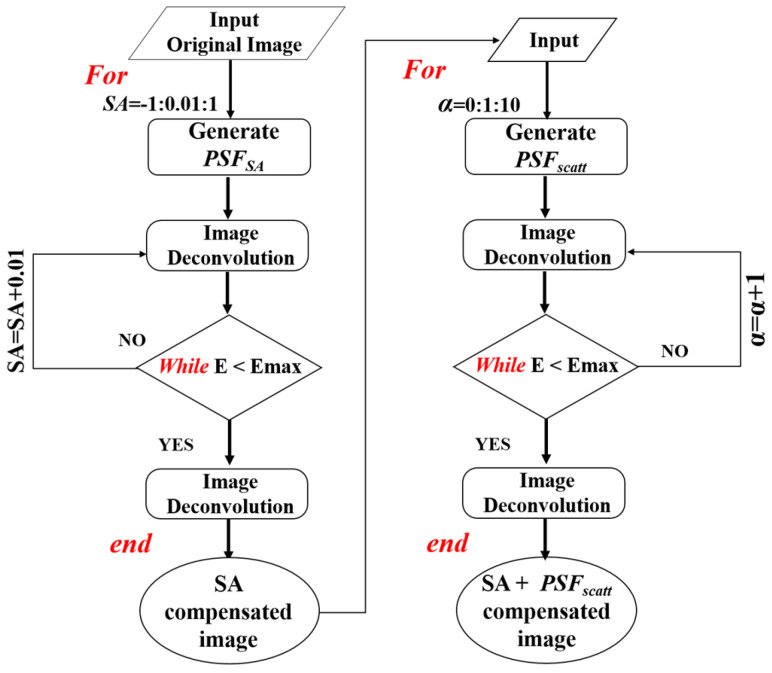
Flowchart describing the deconvolution process (step #2) here developed.

Once the optimal *F_L_* has been found (and therefore the focal plane according to the initial parameters is known), step #2 begins using the image with the optimal *F_L_* as the input image. As depicted in Figure 4, this part of the procedure includes two sequential iterative-optimized deconvolution subroutines intended to compute the *SA* coefficient and the *α* value that maximize the image quality. These will provide the final restored image through the calculation of the optimal *PSF_SA_* and *PSF_scatt_* (i.e., *PSF_SA+scatt_*).

After this deconvolution process, each final image was evaluated by means of the Image Sharpness (*S*) metric defined as

(9) $$S\left(x,y\right)=\sqrt{G_{x}^{2}+G_{y}^{2}}$$

with *G_x_* and *G_y_* being the gradients of the image along X- and Y- directions

The Structural Similarity Index (*SSIM*) (also provided by Matlab^TM^ 2021b Toolbox) computes the similarity between pixels within an image based on neighborhood relationships. Then, the *SSIM* function compares the similarity between a test and a reference image considering three characteristics: luminance, contrast, and structural information.

### 2.5. Samples and Images

Different images were used to test the proposed algorithm. As a proof of concept, an artificial image was firstly used. This corresponded to an image of the 1951 USAF resolution test. This original image was degraded through a convolution operation involving different amounts of *SA* and scattering. The main reasons for chosing an artificial image were the absence of noise sources and the null *SA* and scatterning contributions before the computer-controlled degradation.

The second set of measurements involved images of the human optic nerve head acquired with a confocal scanning laser ophthalmoscope (*CSLO*). The instrument included a polymer dispersed liquid crystal cell (*PDLC*) to acquire images affected by different amounts of scattering. Further details on this setup can be found in [29]. A *PDLC* is a device able to modify its transparency as a function of the voltage. If a null voltage is applied, the internal molecules are randomly orientated, the cell appears cloudy, and the maximum scattering level is produced. When the voltage increases, the amount of scattered light decreases (i.e., *PDLC* transparency increases). For this experiment, *CSLO* images of two young healthy subjects and three scattering levels were used. This were named as *SCL0* (transparent, i.e., maximum voltage applied to the *PDLC*), *SCL1* (moderate scattering level), and *SCL2* (high scattering level).

The last set corresponded to images of different ex vivo ocular tissues recorded with an *AO-MP* microscope [10]: a rat retina (sample #1) and a porcine cornea (sample #2). The microscope used herein incorporates a liquid-crystal *SLM* (used as *AO* element) to compensate for the *SA*. The *AO* algorithm uses a hill-climbing procedure to get the optimum *SA* value. Deconvolved images here obtained were compared to those experimentally acquired using the *AO* procedure. Real images (i.e., the above mentioned three sets of images) were filtered in both spatial and frequency domains, as a pre-processing step before deconvolution, to avoid introducing noise artifacts in the proposed restoration procedure.

## 3. Results

### 3.1. Validation of the Algorithm with Degradation-Controlled Artificial Images: A Proof of Concept

**Figure 5:**
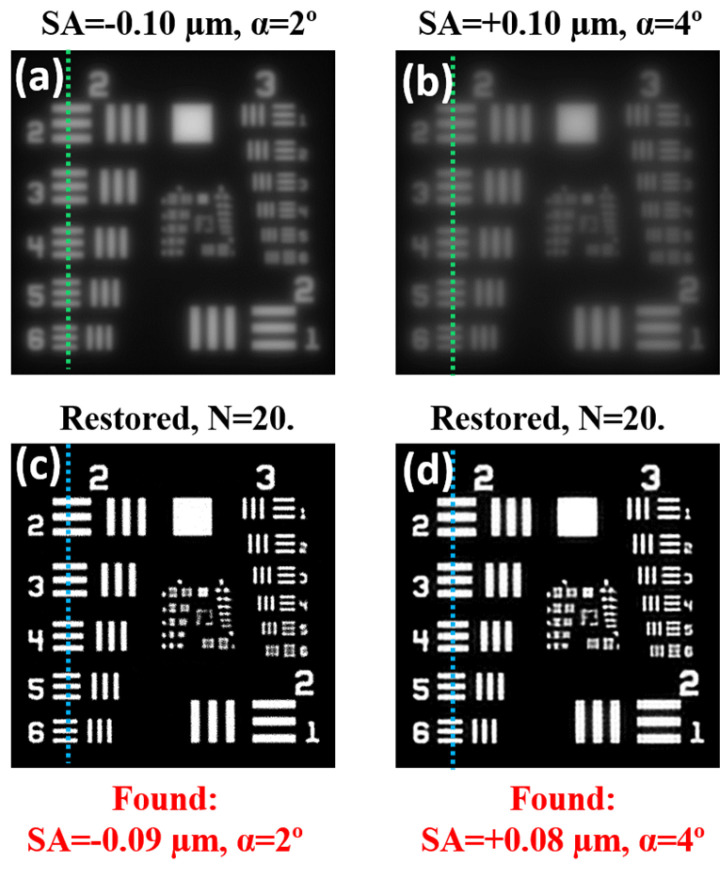
Degraded images combining different amounts of the *SA* and glare angle *α*: (**a**) *SA* = −0.10 μm, *α* = 2°; (**b**) *SA* = +0.10 μm; and *α* = 4°. (**c**,**d**) Corresponding restored images using the deconvolution method here described (*N* = 20). Green and blue dotted lines correspond to the intensity profiles shown in Figure 7.

The original image of the USAF resolution test was degraded through a convolution operation involving two *PSF_SA+scatt_* with different values of *SA* and *α* glare angles. Figure 5a,b shows the corresponding degraded images. The deconvolution method was applied to those images, and the corresponding restored ones are depicted in Figure 5c,d.

**Figure 6:**
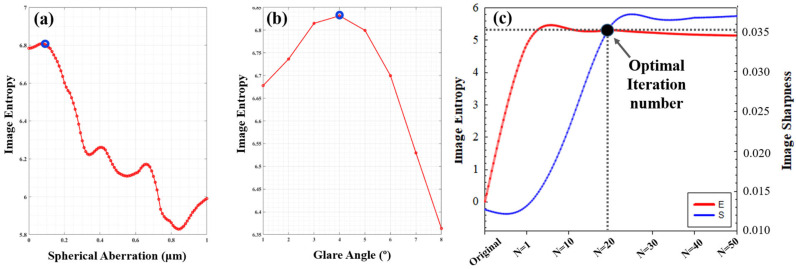
Example of the calculation of *SA* (**a**) glare angle *α* (**b**) values during the deconvolution procedure using E as an image quality metric. (**c**) Experimental determination of the optimal iteration number, *N*. These data correspond to the restoration of image in Figure 5b. Dotted lines intersect the coordinates (*E*,*S*) for the optimal iteration number, *N*.

Details on the restoration process for image in Figure 5b are presented in Figure 6. This shows the evolution of the metric E as a function of the amount of *SA* (Figure 6a) and glare *α* (Figure 6b) during deconvolution. Blue dots represent the values of both parameters (*SA* and *α*) that maximized the metric. For this work, the optimum number of iterations (*N*) during the procedure was chosen as the point where E and S improvement curves intersect (Figure 6c).

Experimentally, the intersection point was found as the threshold from which the optical quality either decreased or did not improve significantly as the number of iterations raises.

Our algorithm can be directly validated by comparing the nominal values of *SA* and *α* used to generate the input degraded images (see legends in Figure 5a,b) and those obtained from the optimization procedure (see red legends in Figure 5c,d). These experimental (nominal) values were −0.09 (−0.10), +0.08 (+0.10) μm for *SA*, and 2° (2°), 4° (4°) for *α*. They agreed well, which corroborates the accuracy of the method here shown.

**Figure 7:**
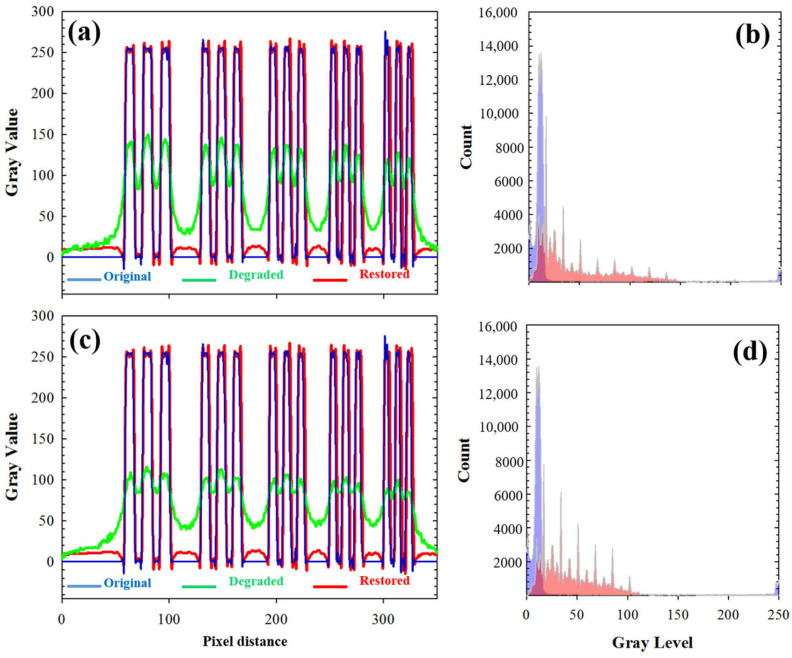
(**a**,**c**) Local intensity profiles along the vertical dotted lines in Figure 5a–d. See text for details. (**b**,**d**) Comparison of the histograms of the degraded (purple) and restored (red) images.

For the sense of completeness, Figure 7a,c depicts the intensity profiles along the vertical dotted lines of Figure 5a–d. Profiles from restored (blue lines) and original images (red lines) did not show statistically significant differences (as shown by a *t*-test). In addition, Figure 7b,d presents the histograms for the original degraded (purple distribution) and restored (red distribution) images. As expected, after deconvolution, an equalization of the histogram was observed, which ensured energy conservation during the operation.

### 3.2. Deconvolution of Human Retinal Images with Induced Scattering

**Figure 8:**
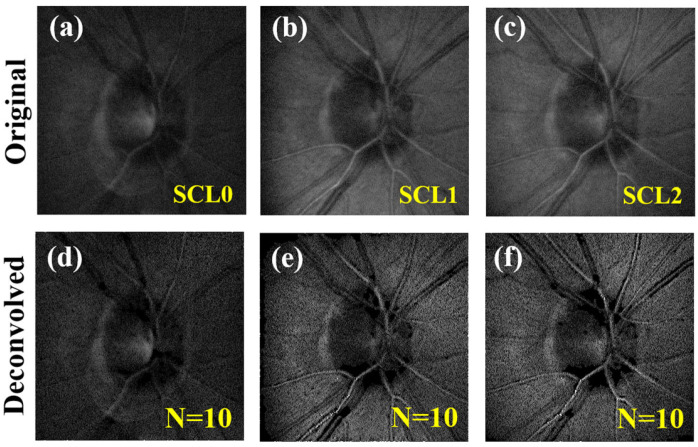
*CSLO* retinal images for three different scattering-induced values (as indicated in the upper images) in subject #1. (**a**–**c**) Original acquired images. (**d**–**f**) Corresponding deconvolved images. Images subtend 15° of visual field.

In this section, the degradation coefficient related to scattering effects (i.e., *α*) was controlled by means of a variable *PDLC*. Upper panels in Figure 8 show, for one of the subjects involved in the experiment, the original *CSLO* retinal images acquired for different scattering-induced levels *SCL0*, *SCL1*, and *SCL2* (see Section 2 for further details). The corresponding restored images after *N* = 10 iterations are presented at the bottom row.

**Figure 9:**
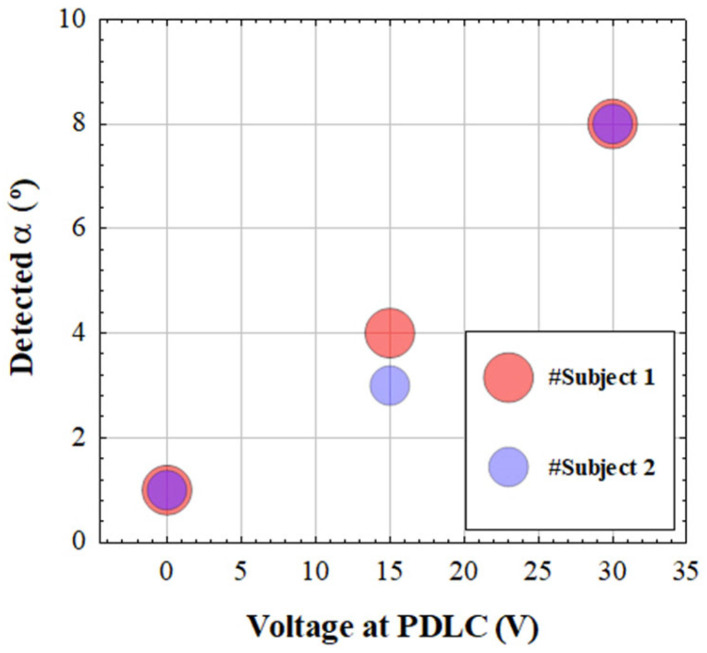
Values of the glare angles *α* obtained through deconvolution as a function of the induced scattering level.

For each experimental scattering condition, Figure 9 shows the glare angles *α* obtained when applying the present algorithm to the *CSLO* images from two subjects. It is interesting to notice that for both subjects, given a scattering-induced level, the computed *α* values were similar. Since the subjects involved were young and healthy, the natural ocular scattering was low [22,23]. Then, the amount of scattering found corresponded exclusively to the induced counter-part, which is coherent with the results found here. The improvements in *E* (S) when comparing final restored and initial images were 97 (176), 101 (165), and 111 (109)% for *SCL0*, *SCL1*, and *SCL2* images, respectively.

**Figure 10:**
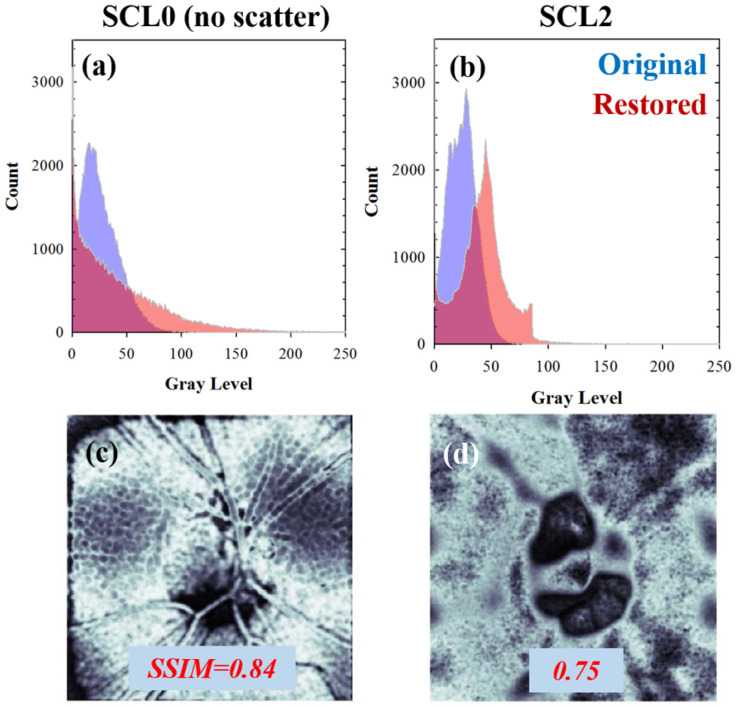
(**a**,**b**) Gray level histograms of the original (purple) and restored (red) *CSLO* images from Figure 8. The corresponding *SSIM* maps (included as a comparison) are scaled between zero (no similarity) and 1 (total similarity) (**c**,**d**).

The results of the deconvolution process can be seen not only in the enhanced image quality (as measured by *E* and *S*) but also in terms of histogram equalization and recovered structural information, as Figure 10 depicts. *SSIM* maps (Figure 10c,d) represent the differences between the original and restored images in terms of image luminance, resolved structure (spatial resolution), and image contrast.

For high scattering levels, fine details and features of retinal images would be hidden by the superimposed veiling glare. If the algorithms properly find and restore this degradation mathematical operator, then the structural differences between original and restored images would impact the computed *SSIM* values, as a greater amount of spatial resolution was recovered, as shown in Figure 10.

### 3.3. Deconvolution of Multiphoton Microscopy Images

It is worth noting that *CSLO* images shown in the previous section were acquired from living human eyes, and therefore the intrinsic *SA* of the human cornea was present (also detected by our algorithm) in the degradation operator that combines *SA* and scattering contributions. In this sense, this section deals with the evaluation of the algorithm with *AO-MP* images of thick scattering biological tissues (see Section 2 above). Further details on this *AO* technique can be seen in [10].

**Figure 11:**
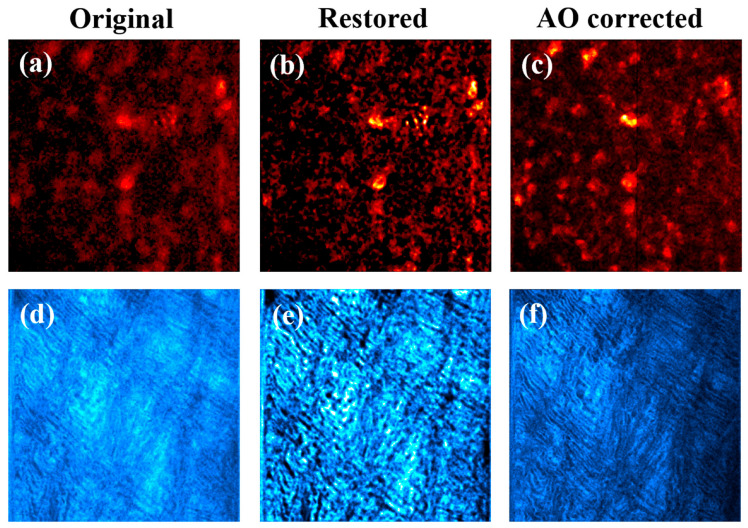
Experimentally recorded MP microscopy images (**a**,**d**) corresponding to a rat retina (**a**) and a porcine cornea (**d**). Restored images using the deconvolution method (**b**,**e**). *AO-MP* images with compensation for *SA* (i.e., *AO* on; right panels (**c**,**f**)). Images within a row share the same color scale. Image size: 210 × 210 μm^2^.

The left column of Figure 11 presents *MP* images acquired in sample #1 (top) and sample #2 (bottom). As expected, these original *MP* images were of limited quality due to the effect of *SA* and scattering. Cells (in Figure 11a) and collagen fibers (in Figure 11d) were hardy distinguished due to the reduced contrast and resolution of the original images. After deconvolution, a noticeable improvement in the images was obtained (middle column).

**Figure 12:**
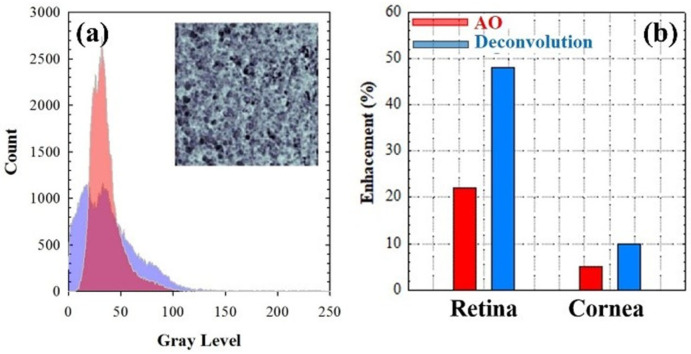
(**a**) Histograms of the *AO-SA* compensated (red) and deconvolution restored (purple) MP images (sample #1) and the corresponding *SSIM* map. (**b**) Enhancement in image quality (*E*) when comparing the original images (Figure 11a,d) and those obtained after *SA* correction (red bars; Figure 11c,f) and deconvolution (blue bars; Figure 11b,e).

For the sense of completeness, the images experimentally recorded through *AO* (i.e., just *SA* compensation) are also depicted (right column). A simple visualization indicates that *MP* images obtained through deconvolution were better that those obtained after using *AO-SA* compensation. This might be mainly due to two reasons: (1) *SA* values differ among both techniques (see Table 1) and (2) our algorithm is also capable of detecting non-negligible *α* values (Table 1) that further improve the quality of the restored images (this scattering was not compensated with experimental *AO*). Those qualitative appreciations are corroborated with the computational results of Figure 12.

Figure 12a compares the intensity histograms of the images using deconvolution (blue) and after the experimental *SA* correction (red). The differences in the *SSIM* values (see map inserted) were mainly due to the uncorrected scattering contribution that remained residual after *AO* operation. Image enhancement (%, in terms of *E*) when comparing the original acquired images and those obtained with *AO-SA* compensation (red bars) and after deconvolution (blue bars) are depicted in Figure 12b.

### 3.4. Contribution of SA and Scattering to Image Quality Degradation

The algorithm here reported takes into account *SA* and scattering, both inherent contributions to the image formation process. This was tested in *CSLO* and *MP* images, where the final restored images were a result of the optimum *SA* and *α* coefficients. Whereas in the former, the amount of induced scattering was controlled, in the latter, only the *SA* was compensated through *AO*. However, *CSLO* images might also be affected by the ocular *SA*, and scattering effects were also present in thick biological tissues during *MP* imaging. In this sense, at this point it is interesting to quantify the individual contribution of every coefficient (*SA* and *α*) to the degradation factor defined in this work.

**Figure 13:**
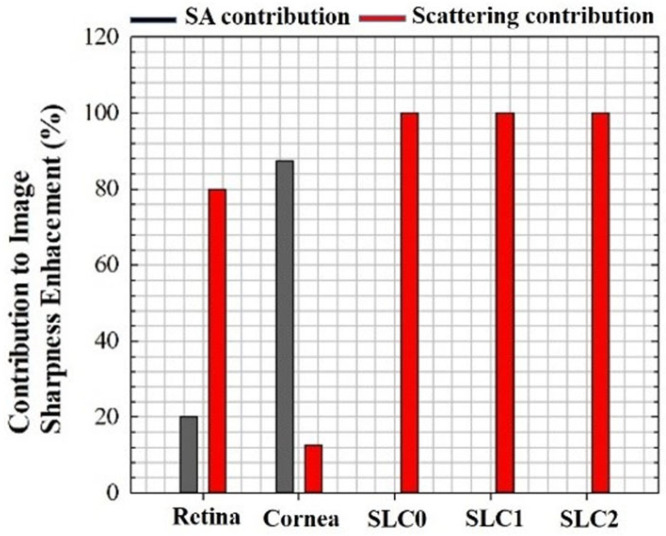
Individual contribution (in %, based on *E* values) of *SA* (black bars) and scattering (red bars) to image enhancement in restored images.

Figure 13 shows the contribution (in %) to the final image enhancement (using S as a metric) after individually compensating *SA* and scattering for different images used in this work. The results show that for *CSLO* images, the *α* coefficient was the dominant contribution to image improvement (i.e., *SA’s* was nearly negigible). On the other hand, for *MP* imaging, the weight of each contribution depended on the sample, that is, the improvement when combining both was better than that corresponding to the use of *SA* or *α* separately.

## 4. Discussion and Conclusions

Image deconvolution algorithms have been widely used to enhance the resolution of blurred images in fluorescence [30] and *MP* microscopy [31,32], retinal imaging [33], and astronomy [34], among others.

Under some experimental conditions, the *PSF* of the optical system required for the deconvolution procedure is unknown. Then, blind (or myopic) deconvolution methods are required to obtain a solution. These are based on aberration compensation. Other previous studies have employed regularization deconvolution to compensate for scattering effects in biological microscopy [35] or diffuse light and halos [36,37] in astronomic images.

Deep learning algorithms based on convolutional neural networks (*CNNs*) have provided new horizons in optical deblurring during the last years [38]; however, those approaches require large image datasets (as well as training processing) that may not be readily available.

However, to the best of our knowledge, blind deconvolution procedures to compensate both aberrations and scattering (or veiling glare) effects have not been reported yet. Unlike previous literature, the present work takes a step forward and merges both effects.

The algorithm incorporates a hill-climbing optimization method that searches for the *SA* and *α* coefficients that optimize the image quality using *E* as a metric. Considering that even in blind deconvolution algorithms a minimal prior knowledge of the *PSF* is required (such as the imposition of the non-negativity of the energy distribution of the deconvolution kernel), the use of the image entropy as the image quality optimization ensures the non-negativity of the estimated *PSF* within the iterative process [39].

As a proof of concept, this was firstly tested in computer-generated images generated by convolving a resolution *USAF* test and *PSFs* with different amounts of *SA* and *α* parametrization (see the accurate performance in Figure 5 and Figure 7). Then, biological images affected by *SA* and scattering were used to evaluate the capabilities of the algorithm. This provided restored images with measurable improvements, where structural details that remained hidden in the original images could be visualized after deconvolution (see for instance Figure 8 and Figure 11).

Our results revealed the proposed algorithm as a useful tool to successfully restore images by accurately obtaining both the *SA* and *α* optimal coefficients. The most interesting finding reported herein is that image enhancement is higher than that reached when using *SA* or scattering compensation separately.

The main limitation of the procedure proposed herein occurs when the spatial resolution of the original image is not enough to obtain an improved image with better entropy. In these cases, the iteration number exceeds the point of optimal convergence (i.e., the intersection *E*,*S*, Figure 6c), and the signal-to-noise ratio increases at the cost of introducing image artifacts as the deconvolution process runs ahead.

To conclude, we developed a blind deconvolution algorithm to compensate for both *SA* and scattering effects. This approach has been carried out defining *PSF_SA_* and *PSF_scatt_* according to the well-established previous publications [21,23]. The algorithm does not require information about the original *PSF* of the optical system used to obtain the image under study. The effectiveness of the approach was demonstrated in microscopy images of different nature.

Future applications of this approach might be of interest in different microscopy imaging applications, ranging from forensic sciences to biomedical environments, in particular in those critical situations where the information on the *PSF* responsible for image formation is not available.

## Figures and Tables

**Table 1:** Comparison of *SA* values for *MP* images in Figure 11 obtained with deconvolution (N iterations as indicated) and *AO*. Values of *α* provided by the algorithm are also shown.

*SA* (µm) [*AO*]	*SA* (µm) [Deconvolution]	*α* (°) [Deconvolution]	*N*
−0.25	−0.36	4	20
−0.10	−0.18	7	30

## Data Availability

The algorithm presented in this study is available on request from the corresponding author.
